# Saliva-based methods for SARS-CoV-2 testing in low- and middle-income countries

**DOI:** 10.2471/BLT.22.288526

**Published:** 2022-10-03

**Authors:** Steph H Tan, Orchid M Allicock, Achilles Katamba, Christine V F Carrington, Anne L Wyllie, Mari Armstrong-Hough

**Affiliations:** a Department of Epidemiology of Microbial Diseases, Yale School of Public Health, 60 College St, New Haven, CT 06510, United States of America (USA).; b Uganda TB Implementation Research Consortium, Makerere University, Kampala, Uganda.; c Department of Preclinical Sciences, University of the West Indies, St. Augustine, Trinidad and Tobago.; d Departments of Social & Behavioral Science and Epidemiology, New York University School of Global Public Health, New York, USA.

## Abstract

As the coronavirus disease 2019 (COVID-19) continues to disproportionately affect low- and middle-income countries, the need for simple, accessible and frequent diagnostic testing grows. In lower-resource settings, case detection is often limited by a lack of available testing for severe acute respiratory syndrome coronavirus 2 (SARS-CoV-2). To address global inequities in testing, alternative sample types could be used to increase access to testing by reducing the associated costs. Saliva is a sensitive, minimally invasive and inexpensive diagnostic sample for SARS-CoV-2 detection that is appropriate for asymptomatic surveillance, symptomatic testing and at-home collection. Saliva testing can lessen two major challenges faced by lower- and middle-income countries: constrained resources and overburdened health workers. Saliva sampling enables convenient self-collection and requires fewer resources than swab-based methods. However, saliva testing for SARS-CoV-2 diagnostics has not been implemented on a large scale in low- and middle-income countries. While numerous studies based in these settings have demonstrated the usefulness of saliva sampling, there has been insufficient attention on optimizing its implementation in practice. We argue that implementation science research is needed to bridge this gap between evidence and practice. Low- and middle-income countries face many barriers as they continue their efforts to provide mass COVID-19 testing in the face of substantial inequities in global access to vaccines. Laboratories should look to replicate successful approaches for sensitive detection of SARS-CoV-2 in saliva, while governments should act to facilitate mass testing by lifting restrictions that limit implementation of saliva-based methods.

## Résumé

La maladie à coronavirus 2019 (COVID-19) continue à affecter les pays à revenu faible et intermédiaire de manière disproportionnée, accentuant le besoin en tests diagnostiques simples, accessibles et fréquents. Dans les endroits disposant de ressources limitées, la détection des cas se heurte souvent au manque de tests disponibles pour le syndrome respiratoire aigu sévère (SARS-CoV-2). Afin de lutter contre les inégalités mondiales en la matière, d'autres types d'échantillons pourraient être exploités, dans le but d'améliorer l'accès au dépistage tout en diminuant les frais qu'il engendre. Les échantillons de salive offrent une méthode de diagnostic fiable, peu invasive et peu coûteuse pour détecter le SARS-CoV-2. Cette méthode est compatible avec le suivi des personnes asymptomatiques, le dépistage des personnes symptomatiques et la collecte d'échantillons à domicile. Les tests salivaires permettent d'atténuer deux problèmes majeurs rencontrés par les pays à revenu faible et intermédiaire: une pénurie de ressources et des soignants surmenés. En outre, les patients peuvent effectuer le prélèvement eux-mêmes et cette méthode nécessite moins de moyens que celle reposant sur l'écouvillonnage. Pourtant, les tests salivaires de détection du SARS-CoV-2 n'ont pas été déployés à grande échelle dans les pays à revenu faible et intermédiaire. Malgré les nombreuses études démontrant l'utilité des tests salivaires dans ces régions, les perspectives d'optimisation de leur mise en œuvre n'ont suscité que peu d'attention. Dans le présent document, nous affirmons que des recherches scientifiques sur leur exécution sont requises pour combler ce fossé entre les faits et la pratique. Les pays à revenu faible et intermédiaire sont confrontés à une multitude d'obstacles dans leurs efforts de dépistage massif de la COVID-19. Et ce, en dépit des profondes inégalités qu'ils subissent dans le monde en matière d'accès aux vaccins. Les laboratoires devraient tenter de reproduire les approches les plus efficaces pour détecter le SARS-CoV-2 dans la salive, tandis que les gouvernements devraient prendre des mesures favorisant un dépistage de masse en levant les restrictions qui entravent le déploiement des tests salivaires.

## Resumen

A medida que la enfermedad por coronavirus de 2019 (COVID-19) sigue afectando de manera desproporcionada a los países de ingresos bajos y medios, crece la necesidad de realizar pruebas de diagnóstico sencillas, accesibles y frecuentes. En entornos de bajos recursos, la detección de casos suele estar limitada por la falta de pruebas disponibles para diagnosticar el coronavirus del síndrome respiratorio agudo grave de tipo 2 (SARS-CoV-2). Para abordar las desigualdades globales en las pruebas, se podrían utilizar tipos de muestra alternativos para aumentar el acceso a las pruebas reduciendo los costes asociados. La saliva es una muestra de diagnóstico sensible, poco invasiva y económica para la detección del SARS-CoV-2 que es apropiada para la vigilancia asintomática, las pruebas sintomáticas y la obtención en el hogar. Las pruebas de saliva pueden reducir dos de los principales problemas a los que se enfrentan los países de ingresos bajos y medios: la escasez de recursos y la sobrecarga de trabajo del personal sanitario. La toma de muestras de saliva permite realizar fácilmente la obtención por cuenta propia y requiere menos recursos que los métodos con hisopos. Sin embargo, las pruebas de saliva para el diagnóstico del SARS-CoV-2 no se han aplicado a gran escala en los países de ingresos bajos y medios. Aunque varios estudios realizados en estos entornos han demostrado la utilidad del muestreo de saliva, no se ha prestado suficiente atención a la optimización de su aplicación en la práctica. En este sentido, se considera que la investigación científica sobre la implementación es necesaria para subsanar esta deficiencia entre la evidencia y la práctica. Los países de ingresos bajos y medios se enfrentan a muchas dificultades en sus esfuerzos por realizar pruebas masivas en relación con la COVID-19, a pesar de las grandes desigualdades en el acceso global a las vacunas. Los laboratorios deberían intentar reproducir los enfoques que han tenido éxito para la detección sensible de la infección por el SARS-CoV-2 en la saliva, mientras que los gobiernos deberían actuar para facilitar las pruebas masivas eliminando las restricciones que limitan la aplicación de los métodos de diagnóstico salival.

## ملخص

مع استمرار تأثير مرض فيروس كورونا 2019 (كوفيد 19) بشكل غير متناسب على الدول ذات الدخل المنخفض والدخل المتوسط، فإن الحاجة تزداد إلى اختبارات تشخيصية بسيطة، ومتواترة، ويسهل الوصول لها. في الأوضاع منخفضة الموارد، غالبًا ما يكون اكتشاف الحالات محدودًا بسبب نقص الاختبارات المتاحة لمتلازمة الجهاز التنفسي الحادة الشديدة فيروس كورونا 2 (SARS-CoV-2). للتعامل مع التفاوتات العالمية في الاختبار، يمكن استخدام أنواع بديلة من العينات لزيادة الوصول إلى الاختبار عن طريق تقليل التكاليف المرتبطة به. اللعاب هو عينة تشخيصية حساسة، قليلة التوغل، وغير مكلفة للكشف عن SARS-CoV-2، وهي مناسبة للمراقبة بدون أعراض، واختبار الأعراض، والتجميع في المنزل. يمكن أن يقلل اختبار اللعاب من تحديين رئيسيين تواجههما الدول ذات الدخل المنخفض والمتوسط، ألا وهما: الموارد المحدودة والعاملين في المجال الصحي المثقلين بالأعباء. يتيح أخذ عينات اللعاب جمعها بشكل ذاتي ومناسب، ويتطلب موارد أقل عن الطرق المعتمدة على المسحة. ومع ذلك، لم يتم تنفيذ اختبار اللعاب لتشخيصات SARS-CoV-2 على نطاق واسع في الدول ذات الدخل المنخفضة والمتوسطة. في حين أن العديد من الدراسات التي تعتمد على هذه الأوضاع قد أثبتت فائدة أخذ عينات اللعاب، إلا أنه لم يكن هناك اهتمام كافٍ بتحسين تنفيذه في الممارسة العملية. نحن نزعم بأن البحث العلمي للتنفيذ ضروري لسد هذه الفجوة بين الدليل والممارسة. تواجه الدول ذات الدخل المنخفض والمتوسطة العديد من العقبات، بينما تواصل جهودها لتوفير اختبار جماعي لفيروس كوفيد 19 في مواجهة التفاوتات الكبيرة في الحصول العالمي على اللقاحات. يجب أن تتطلع المختبرات إلى تكرار الأساليب الناجحة للكشف الدقيق عن فيروس SARS-CoV-2 في اللعاب، بينما يجب على الحكومات العمل لتسهيل الاختبارات الجماعية عن طريق رفع القيود التي تحد من تنفيذ الأساليب المعتمدة على اللعاب.

## 摘要

随着新型冠状病毒肺炎 (COVID-19) 持续对中低收入国家造成严重的影响,对简便、易于频繁使用的诊断检测方法的需在不断增长。在资源匮乏地区,因缺乏可用于严重急性呼吸综合征冠状病毒-2 (SARS-CoV-2) 的检测方法,病例检测也通常会受到限制。为解决全球范围内检测方法不足的问题,可使用替代样本类型来降低相关成本,从而增加检测的使用率。对于 SARS-CoV-2 检测,唾液是一种成本低廉的敏感性微创诊断样本,可用于无症状监测、症状性检测和在家自检。唾液检测可缓解中低收入国家面临的两大挑战:资源有限和医护人员的工作负担过重。唾液取样便于开展自检,并且与基于拭子的方法相比,所消耗的资源也更少。然而,在中低收入国家,通过唾液检测进行 SARS-CoV-2 诊断尚未得到大规模的实施。尽管大量在此类地区开展的研究表明唾液样本很有帮助,但是人们对优化其实际实施的关注度仍不足。我们认为需要对其实施开展科学研究,以弥补循证与实践之间的差距。在全球疫苗获取率严重不足的形势下,中低收入国家在继续推进实施大规模 COVID-19 检测方面仍面临许多障碍。各国的实验室应效仿其他地区在采用唾液样本进行敏感性 SARS-CoV-2 检测中的成功方法,同时各国政府应采取行动解除在实施唾液检测方法方面的限制,以促进大规模检测方法的实施。

## Резюме

Поскольку коронавирусная инфекция - 2019 (COVID-19) продолжает в большей степени поражать страны с низким и средним уровнем дохода, растет потребность в проведении простых, доступных и частых диагностических исследований. В условиях нехватки ресурсов выявление случаев заболевания часто ограничивается отсутствием доступных тестов на наличие коронавирусной инфекции 2-го типа, вызывающей развитие тяжелого острого респираторного синдрома (SARS-CoV-2). Для решения проблемы глобального неравенства в отношении тестирования могут быть использованы альтернативные типы образцов для расширения доступа к проведению тестирования за счет снижения сопутствующих расходов. Слюна является чувствительным, минимально инвазивным и недорогим диагностическим образцом для выявления SARS-CoV-2, который подходит для наблюдения за бессимптомной формой, симптоматического тестирования и сбора в домашних условиях. Анализ слюны может способствовать решению двух основных проблем, с которыми сталкиваются страны с низким и средним уровнем дохода: ограниченности ресурсов и перегруженности медицинских работников. Взятие образцов слюны предполагает удобный самостоятельный сбор и требует меньше ресурсов, чем методы на основе мазков. Однако анализ слюны для диагностики SARS-CoV-2 не был внедрен в широких масштабах в странах с низким и средним уровнем дохода. Несмотря на результаты многочисленных исследований, проведенных в этих условиях, которые продемонстрировали целесообразность взятия образцов слюны, вопросам оптимизации их применения на практике уделялось недостаточно внимания. Для преодоления этого разрыва между фактическими данными и применением на практике необходимы научные исследования в области внедрения. Страны с низким и средним уровнем дохода сталкиваются со многими трудностями, прилагая усилия по проведению массового тестирования на COVID-19 в условиях существенного неравенства в доступе к вакцинам на глобальном уровне. Лабораториям следует стремиться повторить успешные методы для диагностики вируса SARS-CoV-2 в слюне, а государствам следует принять меры для содействия массовому тестированию путем снятия ограничений на использование методов на основе слюны.

## Introduction

In 2020, the Africa Centres for Disease Control and Prevention aimed to increase testing for severe acute respiratory syndrome coronavirus-2 (SARS-CoV-2) from 1300 per million to 16 000 per million population.^1^ They surpassed their goal; by early 2022, over 95 million tests had been performed (about 74 000 tests per million people).^2^ Nonetheless, only one in seven coronavirus disease 2019 (COVID-19) infections were detected in Africa in 2021.^3^ Inadequate community-based testing has likely resulted in thousands of preventable COVID-19-related deaths.^4^ With case detection limited by the low availability of testing, the full extent of COVID-19-related morbidity and mortality in low- and middle-income countries may only later be revealed.^5^

Large-scale diagnostic capacity in lower- and middle-income countries is impeded by the scarcity of biosafety-equipped laboratories to handle samples; the high costs of test materials; the lack of specialized technicians to perform polymerase chain reaction (PCR) assays; and the shortage of trained health professionals to collect nasopharyngeal swab samples.^6^ Low- and middle-income countries also face persistent inequitable access to COVID-19 vaccines – a situation that has been described as vaccine apartheid.^7^ By August 2022, almost 80% of adults in high-income nations had received at least one dose of vaccine.^8^ In contrast, only around 20% of those in low-income countries have received at least one dose.^8^ These overlapping inequities in vaccine accessibility and testing availability must be addressed.

Even as vaccination rates slowly climb in low- and middle-income countries, the need for testing remains high.^9^ A simple, accessible testing strategy is necessary for disease surveillance, enabling public health systems to assess infection prevalence and detect the emergence of variants to prevent further dissemination of the virus.^10^ Moreover, there is a moral imperative to support communities where vaccine availability and access are low. Organizations and government agencies across the globe, especially those of high-income nations, must collaborate to ensure that low- and middle-income countries can access sufficient testing and vaccination resources.

Saliva-based methods can reduce the cost and improve scalability and sustainability of testing programmes in low- and middle-income countries. Saliva has similar effectiveness to nasopharyngeal swabs for detecting SARS-CoV-2,^11^ while also detecting early infection with greater sensitivity than anterior nasal^12^^,^^13^ and mid-turbinate swabs.^14^ This finding has remained true following the rise and worldwide spread of the Omicron variant of SARS-CoV-2.^15^^,^^16^ We discuss here how saliva testing mitigates two major challenges for lower- and middle-income countries: constrained resources and overburdened health workers.

## Test affordability

Swab-based testing approaches are costly and resource-intensive.^17^ In low- and middle-income countries, the cost of swab-based COVID-19 PCR testing can be more than 200 United States dollars (US$).^18^ A single test can cost up to 25% (US$ 210) of the U$ 858 gross domestic product (GDP) per capita in Uganda or 40% (US$ 210) of the US$ 517 GDP per capita in Afghanistan.^19^ While provided at no cost to individuals in some low- and middle-income countries,^18^ testing remains inaccessible to many.

Saliva-based testing approaches are comparatively less costly. As saliva specimens are stable without preservatives and cold-chain management, they do not require expensive, specialized collection tubes.^20^ Saliva-based testing also does not require highly trained health workers, specialized swab supplies or personal protective equipment. The authors of a meta-analysis estimated that saliva collection saves US$ 633 000 for every 100 000 people sampled compared with nasopharyngeal swabs.^21^ Additionally, saliva can be self-collected at clinics and testing centres, thus minimizing discomfort for patients. The method also reduces the risk of occupational exposure to infection for health-care providers,^21^ a particular benefit in lower- and middle-income countries, which have fewer health workers per capita relative to high-income countries.^22^ Self-collection at home can further decrease the human resources needed at testing centres, avoid the need for people to travel and wait at potentially crowded facilities, and reduce the potential for further COVID-19 spread.

Self-collection of saliva is not equivalent to self-testing. Samples may be self-collected under supervision at a testing centre or unsupervised at home to be submitted for laboratory testing. In contrast, self-testing for COVID-19 involves the individual producing their own test result, predominantly using a rapid antigen test.^23^ While these tests can be performed anywhere, including at home, there is no requirement to report the results to health authorities. Although the widespread availability of rapid antigen tests has greatly relieved the pressure on testing sites in high-income settings, the method has led to gaps in the data on the true number of COVID-19 cases.^24^ Thus, self-testing does not contribute to national COVID-19 monitoring and surveillance, whereas self-collection of samples does. Self-collection of saliva remains important to safely expanding a country’s testing efforts.

Another resource-saving approach is the use of methods which bypass the ribonucleic acid (RNA) extraction step (also known as a direct testing approach). These methods have rarely been explored for nasal swabs but are widely validated on saliva samples in large-scale testing programmes.^25^^–^^27^ For example, the Philippine Red Cross launched a saliva-based mass testing programme, providing PCR tests without using RNA extraction^27^ at less than half the cost of other reverse transcription (RT)–PCR tests.^28^ These efforts have enabled more affordable and, importantly, more sustainable testing programmes that have effectively controlled outbreaks.^11^^,^^29^

## Current applications

SARS-CoV-2 saliva testing studies and programmes have been implemented in most regions of the world, including low-cost and less technologically demanding methods that can be adapted to low-resource settings.^29^ Low- and middle-income countries globally have demonstrated the feasibility of saliva-based diagnostics with PCR technology.^29^ In South America, saliva collection has been recommended as a high-performing, non-invasive option. The sensitivity of the method was found to be comparable to that of nasopharyngeal swabs in studies in Brazil,^30^ Chile^31^ and French Guiana.^32^ In South-East Asia, saliva-based testing is considered to be suitable for low-resource areas and able to accurately detect asymptomatic infections through PCR screening, as demonstrated in Viet Nam.^33^ In sub-Saharan Africa, saliva-based PCR testing is offered in several countries, including the Democratic Republic of the Congo and Sudan, while a self-collection programme is currently being piloted in South Africa.^29^

Saliva sampling also allows for sample pooling in laboratories, as several samples can be tested using one reaction. Therefore, the method substantially increases test throughput while reducing the financial and time costs associated with PCR testing.^34^ Pooled saliva testing protocols for SARS-CoV-2 have proven effective in numerous settings globally, such as schools and workplaces in Mexico^35^ and Thailand.^36^ Pooling of samples allows institutions to more rapidly assess infection status and adjust prevention measures as necessary, generating reliable and long-lasting testing programmes that support individuals’ returning to places of work and study.^29^^,^^37^

Loop-mediated isothermal amplification (LAMP) is an alternative diagnostic tool to PCR for low- and middle-income countries because it requires fewer resources, including highly trained laboratory personnel. LAMP is a method that can be used in both laboratory and field conditions, and is compatible with saliva testing.^38^ Saliva-based LAMP can deliver rapid and reliable SARS-CoV-2 test results using simple equipment and processes, involving only a heat block and a single fluid transfer step.^39^ In middle-income countries, LAMP-based testing of saliva was highly sensitive compared with nasopharyngeal swabs in studies in Brazil (78.9%; 60/76 samples),^40^ in China (97.0%; 65/67 samples)^41^ and in Mexico (100.0%; 25/25 samples).^42^ This approach was also an attractive solution when testing options, including government programmes, were scarce in Nicaragua,^29^ where a high prevalence of infection (122/402 people tested; 30.4%) was detected in health-care workers.^43^ The potential for saliva-based LAMP has been demonstrated at scale in the United Kingdom of Great Britain and Northern Ireland, where 86 760 saliva samples underwent direct RT–LAMP.^44^ The method bypasses RNA extraction, demonstrating a sensitivity of 84.6% (209/247 samples) compared with PCR.^44^ Additionally, in the United States of America a workplace surveillance programme using a LAMP method (validated to be 97.0% sensitive; 29/30 samples) tested over 30 000 self-collected saliva samples from 755 individuals in 12 months.^45^

Relative to approaches that require laboratory processing, rapid antigen testing is convenient and inexpensive. Thus, rapid antigen tests are now widespread. For example, in Thailand, the government authorized 63 at-home saliva-based rapid antigen tests (as of early 2022). Rapid antigen tests are also being used for surveillance in Uganda.^29^ However, they are 30.0–40.0% less sensitive than PCR tests.^46^ Moreover, the results of rapid antigen tests taken at home are unlikely to be reported to public health authorities, thus limiting efforts to assess community transmission.

Point-of-care testing platforms could also facilitate rapid, convenient SARS-CoV-2 testing.^47^ Point-of-care platforms are already widely used in low- and middle-income countries for the diagnosis of human immunodeficiency virus (HIV), tuberculosis and viral hepatitis.^47^ Point-of-care testing is decentralized by providing laboratory diagnostic services at or near the site where clinical care is being delivered. In this setting, only a single or few samples can be tested at a time, a strategy that is beneficial for quick determination of infection status in 15–45 minutes.^48^ Although point-of-care testing enables urgent and timely treatment by a health-care provider, the low throughput of the results makes them less useful for surveillance or widescale diagnostics in an outbreak response. Point-of-care testing platforms are proprietary and can also be expensive, which can hinder their accessibility.^49^ However, the ability of point-of-care testing to expand testing capacity, and hence to reduce disease burden and its associated costs in the long-term, is likely to outweigh the implementation costs. When saliva was compared with nasopharyngeal swabs, PCR-based point-of-care testing for SARS-CoV-2 in Italy demonstrated 100.0% specificity (59/59 samples) and 90.2% sensitivity (37/41 samples).^50^ A saliva-based point-of-care testing innovation has also been developed in Türkiye, where an electrochemical immunoassay was developed to enable reliable detection of the SARS-CoV-2 spike S1 protein using a portable device.^51^ Overall, these findings highlight the potential for simplified molecular testing approaches in low- and middle-income countries.

## Improving testing coverage

Dependence on external suppliers and expertise, and competition with higher-income nations for testing resources, has limited the expansion of COVID-19 testing programmes in many low- and middle-income countries.^1^ A less resource-intensive sample type with multiple uses could reduce the coverage gap between lower- and higher-resource countries. Saliva reduces reliance on swabs and other specialized resources, making it a more sustainable option in many settings. Existing workforces and laboratories can be trained to handle saliva through internet-accessible instructions on saliva sample collection and processing.

Crucially, a single saliva sample can simultaneously identify active COVID-19 cases (through SARS-CoV-2 RNA detection) and antibodies that are either vaccine-induced or produced from prior SARS-CoV-2 exposure.^11^ Although serosurveys to monitor COVID-19 burden have been explored in low- and middle-income countries,^52^ saliva is a more convenient sample type that can improve the participation and sustainability of such surveillance programmes. Saliva is less financially burdensome and invasive than the traditional route of requiring a nasopharyngeal swab to detect active infection status or drawing blood for determination of antibody status.^53^ Saliva collection also requires a fraction of the resources needed for drawing blood for antibody detection and can circumvent peoples’ aversion to testing involving needles. Widespread HIV screening in low- and middle-income countries has demonstrated that saliva testing is more acceptable to patients and health workers than serological testing.^54^ With growing support for the use of saliva for SARS-CoV-2 antibody detection,^55^ and given the acceptability of saliva for HIV screening in lower- and middle-income countries, saliva sampling could improve the affordability and acceptability of antibody testing. Thus, saliva sampling for detection of anti-SARS-CoV-2 antibodies could fill testing gaps while allowing population immunity to be monitored.^56^ Combined information on acute and previous SARS-CoV-2 infections is essential for monitoring local outbreaks, determining the proportion of asymptomatic COVID-19 cases and guiding risk mitigation decisions by policy-makers.

Although this new approach is promising, designers of saliva-based COVID-19 testing strategies must be sensitive to the potential impact of such methods on other public health programmes in low-resource settings. Repurposing laboratory facilities for COVID-19 testing can interrupt other essential diagnostic services, including those for HIV, tuberculosis and malaria.^1^ For the first time in a decade, tuberculosis deaths increased in 2020 due to reduced access to diagnostic testing and treatment amidst the COVID-19 pandemic.^57^ Without vigilance, widespread implementation of saliva sampling in low- and middle-income countries could inadvertently disrupt existing medical goods supply chains. For example, if sputum collection containers essential to tuberculosis programmes are diverted to collect saliva samples for COVID-19 testing, timely evaluation of tuberculosis could be compromised.^58^ Ideally, SARS-CoV-2 testing strategies should supplement or be integrated with existing programmes, such as through multiplexing (a laboratory technique that enables the identification of multiple targets in a single diagnostic procedure).

## Discussion

Ultimately, simple, inexpensive and accessible methods of sample collection are critical to expanding and sustaining COVID-19 testing capabilities in low- and middle-income countries. Saliva is simple to self-collect, and the supply chains for mass testing strategies are easier than conventional sample types to establish and sustain. A large body of work has demonstrated that saliva can be a sensitive sample type for SARS-CoV-2 detection and is feasible in low- and middle-income countries.

Wider adoption of saliva as a sample type may be cost-effective as well. In addition to the savings compared with nasopharyngeal swabs, saliva is an ideal sample type for multiplexing. For example, low-cost methods developed for SARS-CoV-2 can be expanded using multiplexing. Saliva-based PCR multiplex approaches enable the non-invasive, simultaneous detection of multiple diseases in a single assay. Consequently, saliva tests could be used to detect and differentiate SARS-CoV-2 RNA from other respiratory pathogens, such as influenza A and B,^59^ and primary pathogens that are endemic in lower- and middle-income countries including HIV,^60^ tuberculosis^61^ and malaria.^62^ Multiplexing could also mitigate the potential interference of COVID-19 testing services with other essential health-care and diagnostic services, such as for tuberculosis and other infectious diseases.

The evidence base for saliva as a reliable and cost-effective sample type for SARS-CoV-2 diagnostics and surveillance is strong and continues to grow.^11^ Nevertheless the adoption, fidelity and sustainability^63^ of saliva-based testing in low- and middle-income countries will depend on the quality of its implementation. When evidence-based practices are transferred to new settings, barriers to implementation can reduce their effectiveness in practice. Implementation science offers systematic approaches to bridging the gap between evidence and practice. Such an approach is needed to optimize delivery of saliva-based COVID-19 testing for low- and middle-income countries. There is immense promise for saliva testing in poorer countries; implementation science can help realize this promise.

With each day, the cooperation between higher and lower income nations to support parallel testing and vaccination efforts becomes more pressing. Low- and middle-income countries face many barriers as they continue to provide mass testing in the face of large inequities in global access to vaccines. Saliva sampling can mitigate some of these barriers by reducing the demand on human and material resources. Laboratories should look to replicate successful approaches for sensitive detection of SARS-CoV-2 in saliva, while governments should act to facilitate mass testing by lifting restrictions that limit implementation of these promising methods.
